# CsPHRs-CsJAZ3 incorporates phosphate signaling and jasmonate pathway to regulate catechin biosynthesis in *Camellia sinensis*

**DOI:** 10.1093/hr/uhae178

**Published:** 2024-06-27

**Authors:** Linying Li, Xueying Zhang, Da Li, Hui Su, Yuqing He, Zelong Xu, Yao Zhao, Yiyi Hong, Qingsheng Li, Ping Xu, Gaojie Hong

**Affiliations:** State Key Laboratory for Managing Biotic and Chemical Threats to the Quality and Safety of Agro-Products, Key Laboratory of Biotechnology in Plant Protection of MOA of China and Zhejiang Province, Institute of Virology and Biotechnology, Zhejiang Academy of Agricultural Sciences, No. 198 Shiqiao Road, Shangcheng District, Hangzhou 310021, China; State Key Laboratory for Managing Biotic and Chemical Threats to the Quality and Safety of Agro-Products, Key Laboratory of Biotechnology in Plant Protection of MOA of China and Zhejiang Province, Institute of Virology and Biotechnology, Zhejiang Academy of Agricultural Sciences, No. 198 Shiqiao Road, Shangcheng District, Hangzhou 310021, China; Institute of Sericulture and Tea, Zhejiang Academy of Agricultural Sciences, No. 198 Shiqiao Road, Shangcheng District, Hangzhou 310021, China; Department of Tea Science, Zhejiang University, No. 886 Yuhangtang Road, Xihu District, Hangzhou 310058, China; Department of Tea Science, College of Horticulture, Henan Agricultural University, No.15 Longzihu University Area, Zhengdong New District, Zhengzhou 450046, China; State Key Laboratory for Managing Biotic and Chemical Threats to the Quality and Safety of Agro-Products, Key Laboratory of Biotechnology in Plant Protection of MOA of China and Zhejiang Province, Institute of Virology and Biotechnology, Zhejiang Academy of Agricultural Sciences, No. 198 Shiqiao Road, Shangcheng District, Hangzhou 310021, China; State Key Laboratory for Managing Biotic and Chemical Threats to the Quality and Safety of Agro-Products, Key Laboratory of Biotechnology in Plant Protection of MOA of China and Zhejiang Province, Institute of Virology and Biotechnology, Zhejiang Academy of Agricultural Sciences, No. 198 Shiqiao Road, Shangcheng District, Hangzhou 310021, China; State Key Laboratory for Managing Biotic and Chemical Threats to the Quality and Safety of Agro-Products, Key Laboratory of Biotechnology in Plant Protection of MOA of China and Zhejiang Province, Institute of Virology and Biotechnology, Zhejiang Academy of Agricultural Sciences, No. 198 Shiqiao Road, Shangcheng District, Hangzhou 310021, China; State Key Laboratory for Managing Biotic and Chemical Threats to the Quality and Safety of Agro-Products, Key Laboratory of Biotechnology in Plant Protection of MOA of China and Zhejiang Province, Institute of Virology and Biotechnology, Zhejiang Academy of Agricultural Sciences, No. 198 Shiqiao Road, Shangcheng District, Hangzhou 310021, China; Institute of Sericulture and Tea, Zhejiang Academy of Agricultural Sciences, No. 198 Shiqiao Road, Shangcheng District, Hangzhou 310021, China; Department of Tea Science, Zhejiang University, No. 886 Yuhangtang Road, Xihu District, Hangzhou 310058, China; State Key Laboratory for Managing Biotic and Chemical Threats to the Quality and Safety of Agro-Products, Key Laboratory of Biotechnology in Plant Protection of MOA of China and Zhejiang Province, Institute of Virology and Biotechnology, Zhejiang Academy of Agricultural Sciences, No. 198 Shiqiao Road, Shangcheng District, Hangzhou 310021, China

## Abstract

Catechins constitute abundant metabolites in tea and have potential health benefits and high economic value. Intensive study has shown that the biosynthesis of tea catechins is regulated by environmental factors and hormonal signals. However, little is known about the coordination of phosphate (Pi) signaling and the jasmonic acid (JA) pathway on biosynthesis of tea catechins. We found that Pi deficiency caused changes in the content of catechins and modulated the expression levels of genes involved in catechin biosynthesis. Herein, we identified two transcription factors of phosphate signaling in tea, named CsPHR1 and CsPHR2, respectively. Both regulated catechin biosynthesis by activating the transcription of *CsANR1* and *CsMYB5c*. We further demonstrated CsSPX1, a Pi pathway repressor, suppressing the activation by CsPHR1/2 of *CsANR1* and *CsMYB5c*. JA, one of the endogenous plant hormones, has been reported to be involved in the regulation of secondary metabolism. Our work demonstrated that the JA signaling repressor CsJAZ3 negatively regulated catechin biosynthesis via physical interaction with CsPHR1 and CsPHR2. Thus, the CsPHRs–CsJAZ3 module bridges the nutrition and hormone signals, contributing to targeted cultivation of high-quality tea cultivars with high fertilizer efficiency.

## Introduction

The tea plant (*Camellia sinensis*) is the main industrial crop cultivated world-wide. Catechins are the major active compounds present in tea. Within the last few years, evidence has revealed that tea catechins confer beneficial effects against several diseases, such as diabetes, cancer and cardiovascular diseases [1–3]. Catechins consist of (−)-catechin (C), (−)-epicatechin (EC), (−)-epicatechin-3-gallate (ECG), (−)-epi-gallocatechin (EGC), (−)-gallocatechin-3-gallate (GCG), and (−)-epi-gallocatechin-3-gallate (EGCG) [4]. Proanthocyanidins (PAs) are polymers or oligomers of flavan-3-ol units such as C, EC, and EGC [5]. The biosynthesis and regulation of these tea catechins has been intensively investigated. The key structural enzymes mainly include chalcone synthase (CHS), chalcone isomerase (CHI), flavanone 3-hydroxylase (F3H), flavonoid 30-hydroxylase (F3′H), flavonoid 3′5′-hydroxylase (F3′5′H), dihydroflavonol 4-reductase (DFR), leucoanthocyanidin 4-reductase (LAR), anthocyanidin synthase (ANS), and anthocyanidin reductase (ANR) [6–8]. Besides, the R2R3-MYB transcription factors, especially CsMYB5 subgroups, control the accumulation of tea catechins in an additive manner by regulating expression of several structural genes [9–11]. For instance, ectopic expression of *CsMYB5c* alters spatial flavonoid and proanthocyanidin accumulation by inducing the expression levels of *ANR*, *CHS*, and *F3′H* [9, 12].

Phosphorus (Pi) is a fundamental element for plants. The limited concentration of inorganic phosphorus in agricultural soil leads to loss of crop productivity [13]. Plants have evolved a variety of sophisticated signal systems to cope with Pi starvation stress. Outstandingly, the acid soil suitable for tea growth is accompanied by phosphorus deficiency [14, 15]. Previous studies have revealed that Pi limitation reduces tea quality and taste due to changes in accumulation of secondary metabolites, such as total catechins, flavonoids, total free amino acids, and theanine [15–17]. However, little was known about the molecular mechanism. In rice and *Arabidopsis*, the complex signaling cascade networks that underlie plant responses to Pi circumstances were discovered through mechanistic analyses [18–23]. In phosphate-deficient conditions PHOSPHATE STARVATION RESPONSE (PHR) transcription factors activate phosphate starvation-inducible (PSI) genes by directly binding to the conserved P1BS element in the promoters to optimize Pi acquisition and utilization. In phosphate-sufficient conditions, the SPX (named after SYG1, Pho81, and XPR1) proteins physically interact with PHRs and negatively regulate the binding affinity of PHRs to the promoters of PSI genes [24, 25]. Conversely, only a few studies have been carried out on woody plants. Researchers identified key genes that respond to Pi starvation in *Populus tomentosa* though transcriptional analysis [26]. Chen *et al*. uncovered the conserved role of PtoPHR1-LIKE3 (PtoPHL3) in regulating Pi acquisition and sustaining Pi homeostasis in poplar though interacting with PtoWRKY40 [27]. Our previous study investigated changes in gene expression in tea under Pi starvation conditions [28]. Nevertheless, the role of SPX–PHR in the regulation of metabolic network in tea leaves needs to be further elucidated.

Jasmonates (JAs) and their derivatives are key phytohormones implicated in stress defense and development [29]. Plants respond to exogenous JA treatment and Pi-deficient conditions through similar changes, such as reduction in primary root elongation, accumulation of anthocyanins and enhancement of plant defense against different stresses [30–33]. The JA signaling pathway has been well researched. Removal of the repressor proteins JAZ (JAZMONATE ZIM DOMAIN**)** allows the core transcription factor MYC2 and other transcription factors to activate the JA response [34]. Moreover, JAZ proteins are widely involved in plant growth and defense though physical interaction with key proteins in other signaling pathways [30, 35, 36]. Our previous study revealed that Pi starvation promoted activation of JA signaling via transcriptional regulation of *OsMYC2* by OsPHR2 in rice [37]. These results indicate that JA signaling is closely linked to phosphorus signaling. However, the roles of the JA pathway in the synthesis of tea catechins and crosstalk with phosphorus signaling remain to be investigated.

In this study, we showed that CsPHR1 and CsPHR2 proteins, master transcription factors in tea, took a role in the biosynthesis of tea catechins induced by phosphorus starvation. CsPHR1 and CsPHR2 activated the transcriptional expression of *CsANR1* and *CsMYB5c* by binding the P1BS *cis*-acting element in promoter regions. We identified the CsSPX1 (TEA018634.1) protein as an interactor to antagonize CsPHR1 activity. More importantly, we demonstrated the physical interaction of CsPHR1/2-CsJAZ3 and linked the JA pathway to the co-regulation of tea catechin synthesis by phosphorus signaling. Collectively, our study reveals the crucial regulatory roles of CsPHR1/2 in modulating the biosynthesis of tea catechins, which provides a mechanistic understanding of how Pi signaling incorporates the JA pathway to regulate the metabolic flux of tea catechins in responding to phosphorus supply.

## Results

### Phosphorus starvation altered catechin accumulation in tea

**Figure 1:**
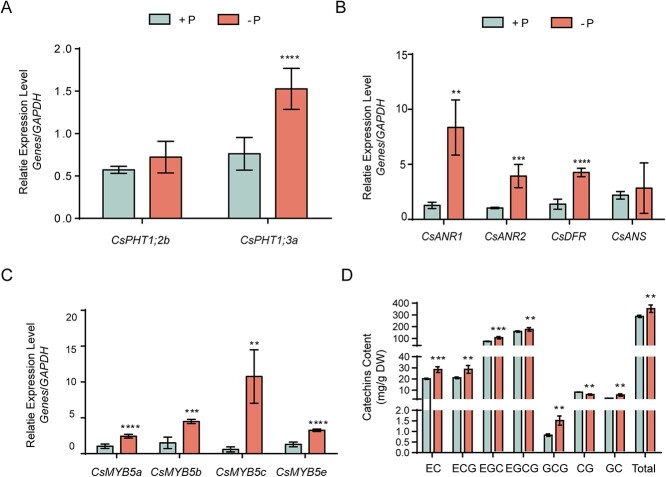
Phosphate starvation induced catechin biosynthetic gene expression and production accumulation. **A**–**C** Relative transcript levels of genes. Young shoots were harvested after Pi-sufficient (+P) or Pi starvation (−P ) treatment for 21 days and used for RT–qPCR. **A** Relative transcript levels of two phosphate transporter genes. **B** Relative transcript levels of key genes in flavonoid biosynthesis. **C** Relative transcript levels of the *CsMYB5* subfamily, which plays key roles in regulating flavonoid biosynthesis. Values are means ± standard deviation (*n* = 6). **D** Accumulation of catechin compounds with Pi starvation (−P ) for 60 days. Values are means ± standard deviation (*n* = 6). ****P < 0.0001, ***P < 0.001, **P < 0.01; Student’s *t*-test compared with samples under +P condition. DW, dry weight.

To test the effect of phosphate starvation on accumulation of tea catechins, 1-year-old Longjing 43 plants harvested from tea plantations were cultured with full-strength nutrient (+P) or Pi-deficient (−P) nutrient solution. During 21-day +P/−P incubation, the responsiveness of PSI genes *CsPHT1;2b* and *CsPHT1;3a* supported the efficacy of the phosphorus deficiency treatment (Fig. 1A). We found that Pi deficiency significantly induced the transcript abundance of synthase genes *CsANR1*, *CsANR2* and *CsDFR*, except for *CsANS* (Fig. 1B). Meanwhile, *CsMYB5a*, *CsMYB5b*, *CsMYB5c*, and *CsMYB5e* acted as regulators of catechins accumulation and were induced by phosphorus starvation in different degrees (Fig. 1C). Furthermore, we detected metabolic changes of tea catechins in response to phosphate starvation. The results showed that the total catechins, including EC, EGC, EGCG, GCG, and GC, increased significantly under Pi starvation stress, except for CG (Fig. 1D). These results indicated that Pi starvation mediated the biosynthesis of tea catechins by regulating expression of synthesis-related genes.

### CsPHRs play important roles in regulating catechin biosynthesis

**Figure 2:**
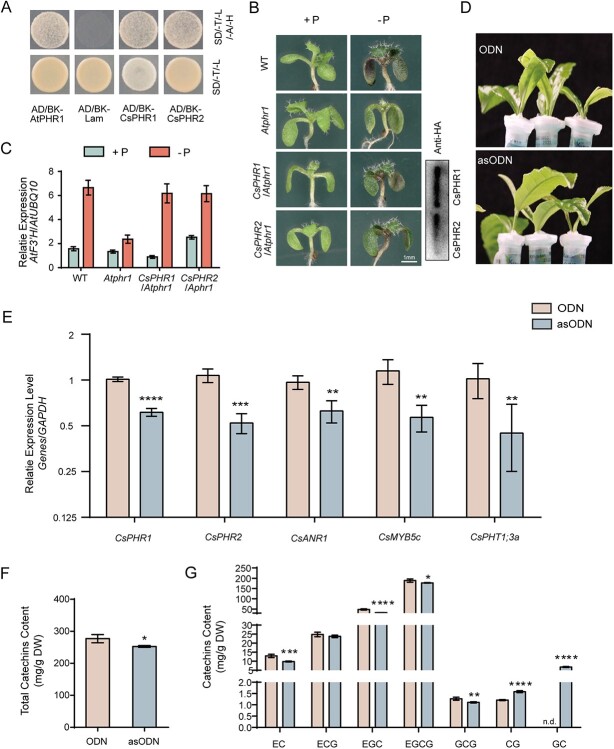
CsPHRs were involved in catechin biosynthesis. **A** Transcriptional autoactivation of CsPHR1 and CsPHR2 in yeast grown on SD/−Leu/−Trp and SD/−Ade/−Leu/−Trp/−His nutrition-deficient medium. Co-expression of BK-AtPHR1 and pGADT7 (AD) served as positive control, and BK-Lam and AD served as negative control. **B**, **C** Anthocyanin phenotyping (scale bar = 1 mm) and *AtF3′H* expression level in Col-0, *phr1*, CsPHR1/*phr1*, and CsPHR2/*phr1* seedlings grown in the Pi-sufficient (+P) or Pi starvation (−P) conditions for 8 days; transcript levels were analyzed by RT–qPCR. Values are means ± standard deviation of three biological replicates. Significant differences were analyzed by ANOVA and Tukey’s multiple comparisons test. **D** Knockdown of *CsPHR*s with antisense oligonucleotide (asODN) solution for 24 h and sense oligonucleotide (ODN) solution as control. **E** RT–qPCR validated the silencing effect of *CsPHR*s and genes that may be regulated by CsPHRs. GAPDH was used as an internal control. Values represent means ± standard error of the mean (*n* = 6). ^****^P < 0.0001, ^***^P < 0.001, ^**^P < 0.01; Student’s *t*-test compared with ODN-treated samples. **F**, **G** Accumulation of total catechins (**F**) and catechin compounds (**G**) in tea leaves treated with asODN or ODN solution. Values represent means ± standard deviation (*n* = 4). ^****^P < 0.0001; ^***^P < 0.001, ^**^P < 0.01, ^*^P < 0.05; Student’s *t*-test compared with ODN-treated samples.

PHRs, as the core transcription factors of the phosphorus signaling pathway, are crucial for controlling the synthesis of flavonoids, but little is known about them in tea plants. According to the sequence of *AtPHR1*, predicted full-length cDNAs of CsPHR1 (TEA002345.1) and CsPHR2 (TEA015819.1) were obtained from the Tea Plant Information Archive (TPIA) (http://tpia.teaplants.cn/). Their deduced protein sequences contained 486 and 478 amino acids, respectively. As with the typical PHR proteins, both of them contained a conserved MYB DNA-binding domain in their N-terminal and a coiled-coil (CC) domain in their C-terminal (Supplementary Data Fig. S1A). The confirmed PHR proteins from *Arabidopsis* (AtPHR1, AtPHL1, AtPHL2, AtPHL3, AtPHL4) [24], rice (OsPHR1, OsPHR2, OsPHR3, OsPHR4) [21], *Triticum aestivum* (TaPHR1-A1, TaPHR1-B1, TaPHR1-D1) [38], *Solanum lycopersicum* (SlPHR1) [37], *Pinellia ternata* (PtPHR1) [39]*, Brassica napus* (BnPHL1) [40], and citrus (CsPHL3) [41] were used to analyze the evolutionary relationship. As presented by the phylogenetic tree, CsPHR1 and CsPHR2 belonged to the same subgroup as SlPHL1, AtPHR1, and BnPHL1, and were more closely related to SlPHL1 (Supplementary Data Fig. S1B). Subcellular localization analysis revealed that CsPHR1-GFP and CsPHR2-GFP localized in the nucleus of *Nicotiana benthamiana* cells when these fusion proteins were expressed under the strong CaMV 35S promoter, and DAPI staining verified this result (Supplementary Data Fig. S1C). RT–qPCR results showed that the expression of CsPHR1 displayed comparatively higher levels than CsPHR2 in different organs of leaves, roots, peels, and fruits but not flowers (Supplementary Data Fig. S1D). Similar to *AtPHR1*, *OsPHR1*, and *OsPHR2* [21], Pi deprivation had no effect on the stable expression of *CsPHR1* and *CsPHR2* in shoot (Supplementary Data Fig. S1E) [21]. An autonomous gene activation test was performed as previously in a yeast system to analyze the transcription autoactivation ability of CsPHR1 and CsPHR2 [42]. Cells with co-expression of CsPHR1 or CsPHR2 with pGADT7 grew well on SD/−Ade/−His /−Leu/−Trp, as the positive control AtPHR1 did (Fig. 2A). Based on the above results, CsPHR1 and CsPHR2 potentially function as transcription activators. In *Arabidopsis*, PHR1 directly regulates the transcription of *AtF3'H* to positively regulate Pi starvation-induced anthocyanin accumulation [13]. We generated *CsPHR1*/*Atphr1* and *CsPHR2*/*Atphr1* plants by introducing CsPHR1/2 fused with HA-tag into the *Atphr1* mutant to further investigate the function of CsPHRs, and the western blot assay demonstrated the successful overexpression of CsPHRs. The reduced anthocyanin accumulation in the *Atphr1* mutant under the Pi deficiency condition could be partly rescued by heterologous expression of CsPHR1 and CsPHR2 (Fig. 2B). The insensitive transcript response of *AtF3′H* in the *Atphr1* mutant under the Pi deficiency condition could be rescued by heterologous expression of *CsPHR1* and *CsPHR2* (Fig. 2C). These results indicated that CsPHR1 and CsPHR2 played homologous roles to AtPHR1 in Pi starvation signaling.

To investigate the function of CsPHRs in catechin biosynthesis in tea leaves, we knocked down both *CsPHR1* and *CsPHR2* using a gene-specific antisense oligonucleotide (asODN). Compared with the controls soaked with sense oligonucleotide (ODN) solution for 24 h, transcript levels of *CsPHR1* and *CsPHR2* were 61 and 49% in the young shoots soaked with asODN solution, respectively. The expression of *CsANR1*, *CsMYB5c*, and the phosphate transporter gene *CsPHT1;3a* was downregulated significantly due to gene silencing of *CsPHR*s, whether under the +P (Fig. 2D and E) or −P condition (Supplementary Data Fig. S2). Interestingly, knockdown of *CsPHR* transcripts also led to significant reduction of total catechins (Fig. 2F). Further analysis revealed that the contents of EC, EGC, EGCG, and GCG were positively correlated with the expression of *CsPHR*s, whereas the contents of CG and GC showed the opposite (Fig. 2G). These results indicated that CsPHRs were involved in changes of tea catechins under Pi starvation though regulating the expression of genes related to catechin biosynthesis.

### The SPX–PHR module regulated catechin biosynthesis by modulating the transcription of *CsANR1* and *CsMYB5c*

**Figure 3:**
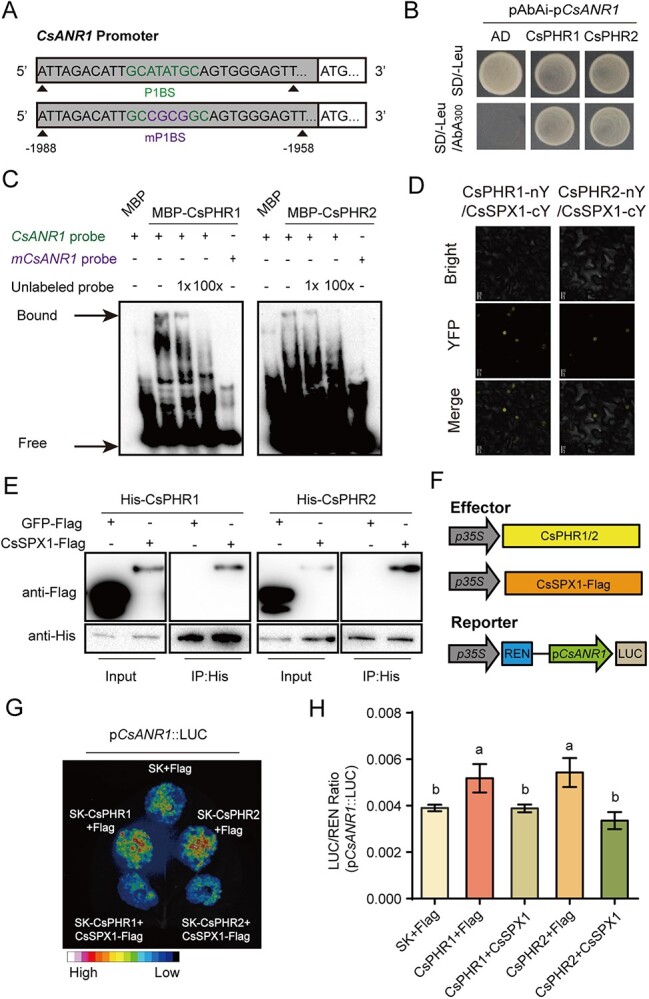
CsSPX1 suppresses the function of CsPHRs in binding the P1BS element of *CsANR1* and *CsMYB5c*. **A** Diagram of the wild-type P1BS element and four base-mutated P1BSs (mP1BSs) in the *CsANR1* promoter. **B** Y1H analysis of CsPHRs binding the promoter of *CsANR1*. Empty pGADT7 vector was used as negative control. Yeast cells containing different plasmid combinations were grown on the selective medium SD−Leu with 300 μm aureobasidin A (AbA). **C** EMSA results indicated that CsPHR proteins bind to the *CsANR1* promoter. Arrows show CsPHRs-bound or free DNA. The competitive protein–DNA binding assay was performed with an increasing amount of unlabeled DNA probe (1- and 100-fold). **D** Analysis of CsSPX1 and CsPHR interaction by BiFC. Confocal images of *N. benthamiana* epidermal cells expressing different construct combinations as indicated. Scale bar = 20 μm. **E**  *In vitro* semi-pull-down assays showing the interaction between CsSPX1 and CsPHRs. **F** Schematic diagram of the luciferase reporter system. **G**, **H** Transactivation assays in *N*. *benthamiana* leaves. **G** Luciferase intensity was imaged 48 h after infiltration. **H** LUC/REN activity obtained from co-transfection with the indicated reporter constructs or empty effector constructs, Values are means ± standard deviation of three biological replicates. Different letters (a and b) indicate significant difference (ANOVA, Tukey’s multiple comparisons test, *P* < 0.05).

Previous studies have revealed that PHRs from other species positively regulated the Pi starvation response by binding the P1BS *cis*-acting element in promoter regions [43]. Bioinformatics analysis showed that a P1BS *cis*-element existed in the 2-kb promoter region of *CsANR1* and *CsMYB5c* (Fig. 3A and Supplementary Data Fig. S3A), but P1BS elements were absent from the promoters of other genes shown in Fig. 1. The yeast one-hybrid assay (Y1H) demonstrated that both CsPHR1 and CsPHR2 could bind to the P1BS element in the 2-kb length *CsANR1* promoter (Fig. 3B). To rule out false positives, we constructed the reporter with a 0.5-kb length promoter sequence of *CsANR1* that contained P1BS, and CsPHR2 also bound to the 0.5-kb length *CsANR1* promoter (Supplementary Data Fig. S4). The electrophoretic mobility shift assay (EMSA) was then performed with MBP-fused CsPHR proteins and DNA fragments containing the P1BS from *CsANR1. In vitro*-translated MBP was used as negative control. A mobility shift for biotin-labeled *CsANR1* probes was observed in the presence of MBP-CsPHR1 and MBP-CsPHR2 but not MBP. A competition assay using mutant version or unlabeled probes with incremental concentration confirmed the specific binding (Fig. 3C).

**Figure 4:**
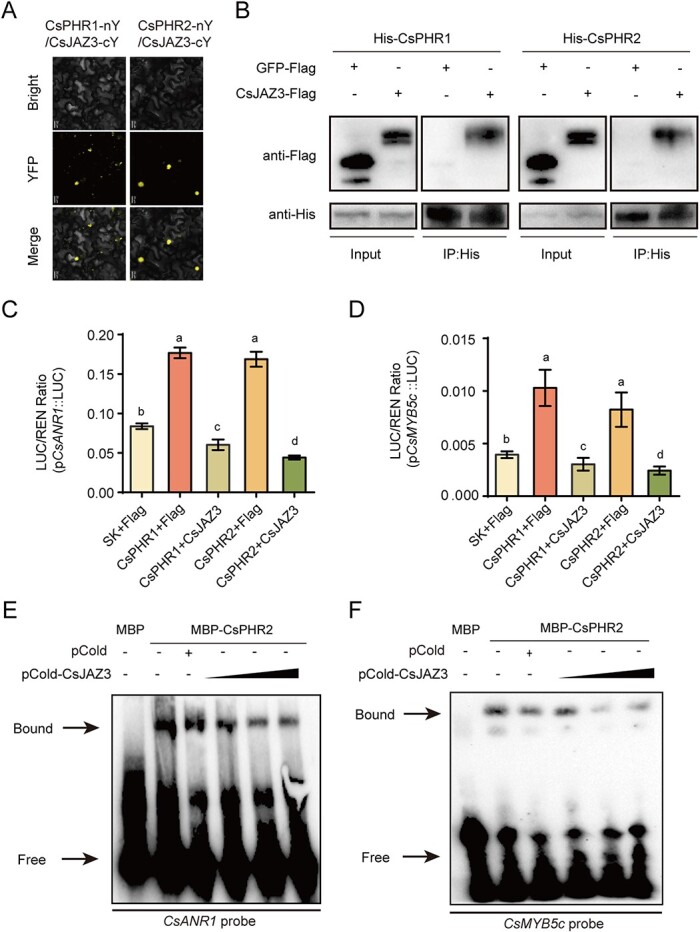
Function of CsJAZ3-CsPHRs transcriptional regulatory module in tea catechin biosynthesis. **A** Analysis of CsJAZ3 and CsPHRs interaction by BiFC. Confocal images of *N. benthamiana* epidermal cells expressing different construct combinations as indicated. Scale bar = 20 μm. **B**  *In vitro* semi-pull-down assays showing the interaction between CsJAZ3 and CsPHRs. **C**, **D** LUC/REN activity obtained from co-transfection with the indicated reporter constructs or empty effector constructs. Values are means ± standard deviation of three biological replicates. Different letters (a–d) indicate significant difference (ANOVA, Tukey’s multiple comparisons test, *P* < 0.05). **E**, **F** EMSA showed that the CsJAZ3–CsPHR2 interaction attenuated the DNA binding activity of CsPHR2 to the P1BS motif of *CsANR1* (**C**) and *CsMYB5c* (**D**) in the promoter. The triangle shows the rise in pCold-CsJAZ3 protein concentration from 50 to 150 ng. Signs - and + indicate the absence and presence of the corresponding proteins, respectively.

To determine how CsPHRs regulate the expression of *CsANR1 in vivo*, a transient expression assay was carried out in which CsPHR1 or CsPHR2 was co-expressed with the *CsANR1* promoter driving the luciferase reporter in tobacco. Our results demonstrated that CsPHR1 and CsPHR2 activated the activity of *CsANR1* promoter. Compared with the control vector, the presence of CsPHR1 and CsPHR2 significantly increased the *pCsANR1*::LUC activity (Fig. 3G and H). Also, CsPHR1 and CsPHR2 directly targeted the DNA promoter of *CsMYB5c* (Supplementary Data Fig. S3B and C) and activated its transcription activity (Supplementary Data Fig. S3D). These findings indicated that both CsPHR1 and CsPHR2 were key regulators involved in Pi deficiency-induced catechin biosynthesis.

According to the earlier findings, the host SPX–PHR transcriptional regulatory module is a key player in modulating phosphate homeostasis in rice and *Arabidopsis* [18]. Bimolecular fluorescence complementation (BiFC) assays verified the interaction between CsSPX1 and the two CsPHRs in the nucleus of tobacco cells (Fig. 3D). No fluorescence signal was detected in the control groups (Supplementary Data Fig. S5A). Next, immunoblot analysis showed that CsSPX1 protein expressed in *N. benthamiana* leaves could be pulled down by MBP-CsPHR1 and MBP-CsPHR2 *in vitro*, but not the control GFP protein (Fig. 4E). In line with previous studies, the CsPHR-mediated augmentation of LUC activity was strongly suppressed when CsSPX1-Flag and SK-CsPHR1 or SK-CsPHR2 were co-expressed with the *pCsANR1*::LUC reporter (Fig. 3E), whereas CsSPX1 alone had no effect on *pCsANR1*::LUC activity (Supplementary Data Fig. S5B). Our results suggested that CsSPX1 negatively regulated CsPHR-mediated catechin biosynthesis.

### CsJAZ3 interacted with and inhibited transcriptional activity of CsPHRs

JAZ repressors of the JA signaling pathway have been reported to interact with various transcription factors and inhibit their transcription activities [44, 45]. We wondered whether this specific interaction existed in tea. Our speculation was confirmed by the BiFC assay using tobacco leaves. A fluorescence signal in cells expressing CsJAZ3-cYFP/CsPHR1-nYFP or CsJAZ3-cYFP/CsPHR2-nYFP was observed in the nucleus, separately (Fig. 4A). No fluorescence signal was detected in the control groups (Supplementary Data Fig. S6A). We then performed the semi-pull-down assay to verify the interaction *in vitro*. Immunoblot analysis showed that both MBP-CsPHR1 and MBP-CsPHR2 could pull down the CsJAZ3 protein expressed in leaves of *N. benthamiana*, but not the control GFP protein (Fig. 4B).

The transient transactivation assay was performed to further test the effect of CsJAZ3 on transcriptional activity of CsPHRs. Luciferase activity of *pCsANR1*::LUC and *pCsMYB5c*::LUC was tested in *N. benthamiana* leaves co-expressing CsPHR1/2 and CsJAZ3. We found that the presence of CsJAZ3 would reduce the activation of CsPHR1/2 on p*CsANR1*::LUC (Fig. 4C) and p*CsMYB5c*::LUC (Fig. 4D). Additionally, we found that CsJAZ3 alone also inhibited the activity of *pCsANR1*::LUC and *pCsMYB5c*::LUC (Supplementary Data Fig. S6B), which may be attributable to the changes in the outputs of JAs caused by CsJAZ3 ectopic expression. CsPHR2 was selected to analyze the mechanism by which CsJAZ3 inhibited the transcriptional activity of CsPHRs. The EMSA assay further supported that CsJAZ3 significantly reduced the binding affinity of CsPHR2 to the promoter of *CsANR1* (Fig. 4E) or *CsMYB5c* (Fig. 4F) in a dose-dependent manner. These results demonstrated that the CsJAZ3–CsPHRs transcriptional regulatory module played an important role in tea catechin biosynthesis.

### Methyl jasmonate induced catechin biosynthesis in a CsPHR-dependent manner

**Figure 5:**
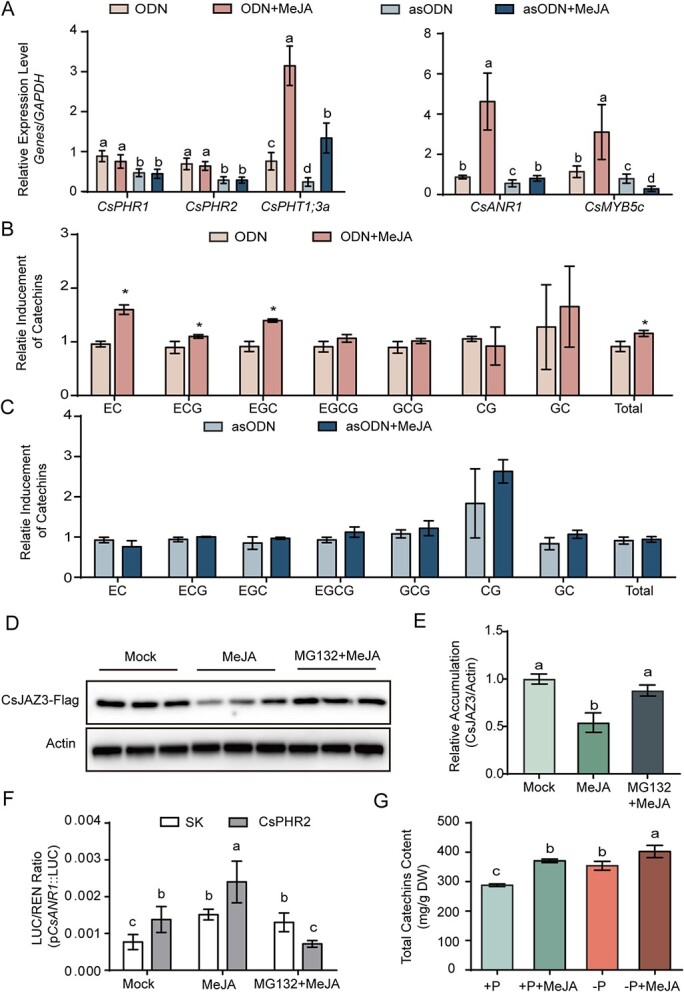
CsPHRs integrate Pi and JA signaling to regulate catechin biosynthesis. **A** RT–qPCR validated the silencing effect of *CsPHR*s and genes that may be regulated by CsPHRs. GAPDH was used as an internal control. **B** Relative inducement of catechins in tea leaves treated with ODN solution and MeJA. **C** Relative inducement of catechins in tea leaves treated with asODN solution and MeJA. **D**, **E** Representative western blot showing the accumulation of CsJAZ3-Flag in *N. benthamiana* leaves treated with 25 μM MG-132 or in the presence or absence of 100 μM MeJA. Protein levels were normalized against actin levels. **F** Transactivation assays. Luciferase (LUC)/*Renilla* (REN) activity obtained from co-transfection with an empty effector (SK) and the indicated reporter (CsPHR2) construct. Leaves were infiltrated with MG132 (10 μΜ) at least 12 h prior to harvesting. Exogenous MeJA (100 μM) was rubbed onto *N. benthamiana* leaves and fluorescence was measured after 4 h. **G** Accumulation of total catechins in young shoots with Pi starvation and MeJA treatment. Data are means ± standard error, and different letters indicate significant differences at *P* < 0.05 tested by ANOVA.

The repression of CsPHRs by CsJAZ3 also supported the methyl jasmonate (MeJA) induction of the catechin synthase genes [46]. Our RT–qPCR results also demonstrated that MeJA treatment induced the expression of *CsANR1*, *CsANS*, and *CsMYB5c* (Supplementary Data Fig. S6C). To investigate the effect of CsJAZ3–CsPHR1/2 interaction on MeJA-mediated catechin biosynthesis, tea leaves soaked with ODN or asODN solution were treated with 10 μM MeJA. The decreased expression levels of *CsPHR1* and *CsPHR2* indicated effective knockdown in tea leaves. MeJA triggered the transcriptional abundance of *CsPHT1;3a*, *CsANR1*, and *CsMYB5c* in tea leaves soaked with ODN solution. Notably, knockdown of *CsPHR1* and *CsPHR2* attenuated JA-induced transcriptional expression of *CsPHT1;3a*, *CsANR1*, and *CsMYB5c* to a great extent (Fig. 5A). In the presence of MeJA, total catechins of tea leaves soaked with ODN solation were increased by 1.3-fold. Among them, EC, the product of CsANR1, was induced significantly by 1.7-fold. Besides, the contents of ECG, EGC, and EGCG increased in response to MeJA treatment (Fig. 5B). On the contrary, the MeJA-induced catechin accumulation was not observed in tea leaves in which *CsPHR1* and *CsPHR2* were silenced (Fig. 5C). These results suggested the involvement of *CsPHR1/2* in JA signaling. Previous investigations have shown that the 26S proteasome-specific inhibitor MG132 blocks the JA-mediated degradation of JAZ proteins [33, 47]. Immunoblotting results revealed that MG132 inhibits JA-induced CsJAZ3 protein degradation, suggesting that CsJAZ3 acted as a repressor of JA signaling in tea (Fig. 5D and E). We then investigated MeJA-mediated regulation of *CsANR1* by transiently expressing effector–reporter constructs in *N. benthamiana* leaves. As shown in Fig. 5F, MeJA treatment further boosted the transcriptional activation of CsPHR2 on the *CsANR1* promoter; moreover, this effect was abolished in leaves infiltrated with MG132. In addition, Pi insufficiency and MeJA synergistically promoted catechin biosynthesis (Fig. 5G) also suggesting JA induced catechin accumulation though interactions of CsJAZ3 and CsPHRs. Collectively, these findings revealed that the crosstalk between JA and Pi signaling was mediated by the CsJAZ3–CsPHR1/2 module, which was crucial for the precise regulation of catechin biosynthesis in *C. sinensis*.

## Discussion

It is well known that Pi deficiency induces the catechin biosynthesis pathway in plants [48–50]. In this study, we found that Pi starvation increased the contents of EC, ECG, EGC, EGCG, GCG, and GC in young shoots of Longjing 43 (Fig. 1). Previously, the accumulation of total catechins in response to Pi deficiency was researched in the Zhongcha 108 cultivar [51]. However, the duration of incubation, tea cultivar, and other environmental conditions employed in Pi deficiency treatments would alter the change in content and composition of catechins. For instance, the contents of EGCG, EGC, and GC increased under the Pi starvation condition in the Fengqing cultivar, whereas those of EC and EGC were induced in the Longjing 43 cultivar [15].

The change in secondary metabolites in tea leaves in response to Pi deficiency has received wide coverage [15, 52, 53]; however, the mechanism is still not clear. Here we isolated two potential transcription factors of the phosphorus signaling pathway, CsPHR1 and CsPHR2, that contain the MYB and CC domains in tea. The differential expressions of *CsPHR1* and *CsPHR2* in various organs indicated their different roles in the growth and development of tea plants. Both of them were nuclear-localized proteins, which was important for their function as transcription factors. Indeed, CsPHR1 and CsPHR2 showed strong transactivation activities *in vivo* (Fig. 2). Knockdown of *CsPHR*s led to a lower transcriptional abundance of the phosphate transporter gene *CsPHT1;3a* (Fig. 3B). Taking these results together, CsPHR1 and CsPHR2 shared the conserved characteristics of core transcription factors in the phosphorus signaling pathway. In addition, the function of CsPHR1 in regulating flavonoid accumulation was redundant with respect to CsPHR2.

Tea catechins are produced following the flavonoid metabolic pathways, regulated by multiple enzymes and transcription factors [9, 54]. We found that a P1BS motif located on the promoter region of *CsANR1*, which catalyzes the final two steps of flavonoid biosynthesis, and resulting in the creation of EC and EGC [15, 55]. Since CsANR1 was responsible for the synthesis of EC and EGC, Pi deficiency induced the gene expression of *CsANR1* along with its catalytic products (Fig. 1). Conversely, the knockdown of CsPHRs led to the downregulation of *CsANR1* as well as decreases in the contents of EC and EGC (Fig. 3). Further experiments verified that both CsPHR1 and CsPHR2 specifically targeted the P1BS motifs in the promoter of *CsANR1* to activate its expression. These results indicated that CsPHR proteins induced the expression of structural genes in flavonoid synthesis, thereby enhancing tea catechin accumulation in young shoots of Longjing 43. In *Arabidopsis*, PHR1 directly recognizes and binds the P1BS motif in the promoter regions of genes *F3'H* and *LDOX* to modulate Pi starvation-induced anthocyanin accumulation [13]. Therefore, the induction of flavonoids by Pi deprivation is conserved, whereas the structural genes regulated by PHRs in different species are specific. In *Arabidopsis*, in addition to directly regulating structural genes, Pi starvation also influences conserved regulators to indirectly alter anthocyanin accumulation. For instance, our previous study demonstrates that AtSPX4 interacts with AtPAP1, a core factor that activates anthocyanin biosynthesis, to regulate expression of *AtDFR* and anthocyanin accumulation [49]. In this study, we also found that CsPHR1/2 not only regulated the structural enzyme gene *CsANR1*, but also directly regulated the transcription factor *CsMYB5c*. Ectopic expression of *CsMYB5c* in *N. tabacum* significantly enhanced the content of catechins, including C, EC, and EGC [9, 12]. This means that Pi signaling could directly regulate the whole metabolic flow. This may also explain the low Pi adaptation in tea plants. In addition, CsSPX1 interacted with CsPHRs and negatively regulated the function of CsPHRs (Fig. 5), which is consistent with other plant species [20, 56–58], indicating that the modulation of plant secondary metabolites by the SPX–PHR regulatory circuit is conserved.

**Figure 6:**
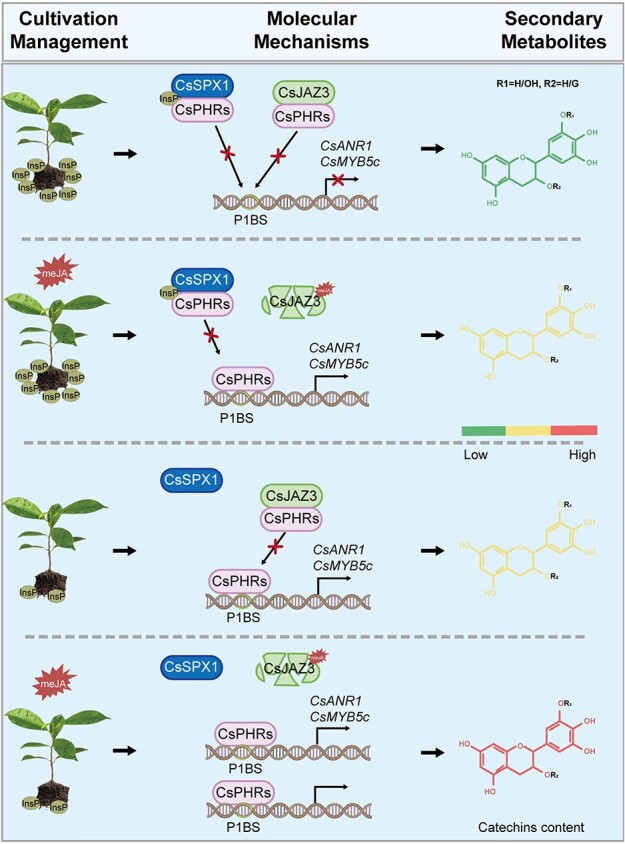
A model for CsPHRs-mediated catechin biosynthesis in tea. In the phosphate-sufficient condition, CsSPX1 interacts with CsPHRs to inhibit the transcriptional activity of CsPHRs on *CsANR1* and *CsMYB5c*, and thus negatively regulates Pi-mediated catechin accumulation. In the presence of MeJA, degradation of CsJAZ3 via the 26S-proteasome pathway releases CsPHRs from physical interaction to activate Pi-mediated catechin accumulation. When phosphate levels are low and MeJA is present, CsPHRs are liberated from CsSPX1 binding and CsJAZ3 degradation, which promotes the production of additional tea catechins.

JA and JA-induced secondary metabolites are related to plant broad-spectrum resistance to unfavorable conditions, such as drought, cold, and pathogens [26, 59]. Our previous report revealed that Pi starvation activates JA signaling and enhances resistance of rice to *Xanthomonas oryzae* pv. *oryzae* [37]. Similarly, Pi deficiency induces the jasmonate pathway, promoting the resistance of *Arabidopsis* to insect herbivory [31] and resistance of cotton to *Verticillium dahliae* [48]. Phytohormone analysis showed that Pi deficiency significantly induced the accumulation of MeJA in tea shoots, but produced no significant change in JA and JA-Ile contents (Supplementary Data Fig. S7). The results are consistent with our research on rice [37]. These findings suggest that when faced with challenging conditions like nutrient scarcity, plants coordinate the JA pathway to enhance the adaptability.

JAZ degradation caused by MeJA treatment releases many transcription factors to regulate various physiological processes [34, 35]. In *Arabidopsis*, PHR1 interacts with JAZ and MYC2 to maintain the homeostasis of JA signaling under Pi starvation stress [60]. It has also been reported that OsJAZ11 regulates Pi homeostasis via physical interaction with OsSPX1, whereas OsJAZ11 does not influence the inhibitory effect of OsSPX1 on OsPHR2 activity [30]. Different from earlier studies, our work on tea plants discovered that the JA pathway regulates Pi starvation-induced secondary metabolite accumulation via physical interaction of CsJAZ3 and CsPHR1/2 (Fig. 6). This crosstalk between Pi signaling and JA hormone could be a mechanism of adaptation to adversity for tea plants. Tea grows chronically in a low phosphorus environment [14, 15]. The function of CsPHR1/2 proteins is suppressed not only by CsSPX1, but also by CsJAZ3. In this way, CsPHR1/2 is still partially inhibited even under low Pi conditions, which reduces the waste of energy and nutrition (Fig. 6). This CsPHR1/2-CsJAZ3 module also explains the absence of a purplish red phenotype in young shoots of tea under low Pi conditions, unlike *Arabidopsis* [13]. Taken together, our results uncover a pivotal molecular mechanism involving linkage between soil nutrients and crop quality, providing a strategy for crop management.

## Materials and methods

### Plant materials, growth conditions, and experimental treatments

Full-strength (+P) nutrient solution (Coolaber, NSP1030) with KH_2_PO_4_ (8.53 mg/l) that supports Longjing 43's hydroponic growth [61] was used to cultivate tea plants, and Pi-deficient (−P ) nutrient solution (Coolaber, NSP1030-P) without KH_2_PO_4_ was used in the treatment group and changed once a week. One-year-old seedlings of Longjing 43 were transplanted from the field to the greenhouse. The plants were cultivated in plastic pots containing 10 l aerated nutrient solution for 2 months under a 10 h/14 h light/dark photoperiod at room temperature (25 ± 1°C). Young shoots that reached the desired developmental stage for harvesting were sampled and quickly frozen in liquid nitrogen for further analysis.

MeJA treatment was carried out as previously [62], and leaves for RT–qPCR analysis were harvested after MeJA treatment for 3 h with three repetitions. Seventy-two individual tea plants treated with +P/−P solution for 60 days were evenly sprayed with 50 ml of 100 μM MeJA and sprayed once a day. Fresh tea leaves were plucked for analysis of catechin compounds after MeJA treatment for 7 days. After being sprayed with 200 μl of ethanol that had been dissolved in 50 ml of 0.1% Triton X-100 solution, the control groups (+P and −P ) underwent the same processing as the MeJA-treated samples.

*Arabidopsis* seeds were germinated on Murashige and Skoog medium with (+P, Caisson Labs, MSP01) or without phosphate (−P , Caisson Labs, MSP11) containing 0.05% sucrose at 22°C under a 14 h light/10 h dark cycle. The anthocyanin phenotype was captured by a super-depth-of-field microscope (Keyence, VHX-950F).

### Extraction and quantification of catechins

A modified protocol was used to determine the catechin content [62, 63]. Tea leaves were dried for 4 h in an oven at 80°C and then ground into powder. One milliliter of a 50% (v/v) ethanol solution was used to extract the sample powder (0.015 g) at 70°C for 30 min. The supernatant was submitted to HPLC for analysis of catechin compounds after centrifugation (4°C, 12 000 rpm, 10 min).

### Isolation of CsPHRs and sequence alignment

Sequences of *CsPHR1* and *CsPHR2* were identified from the Tea Plant Information Archive (TPIA) using the sequence of *AtPHR1* as the query. Subsequently, leaf cDNA of Longjing 43 was used as the template to amplify them with specific primers (Supplementary Data Table S1). MEGA11 software was used to examine multiple sequence alignment of the PHRs. Then the neighbor-joining method was used to estimate the phylogenetic tree connecting the tea plant to other species with 1000 bootstrap replicates.

### RNA extraction and RT–qPCR

Total RNA of various tissues from Longjing 43 was purified using TRIzol Reagent (Invitrogen, USA). The ABI QuantStudio 6 Flex (Applied Biosystems, USA) was used to perform RT–qPCR with SYBR qPCR Mix (GenStar, A304). *GAPDH* was used as an internal control. Primers used for RT–qPCR are listed in Supplementary Data Table S1.

### Silencing of CsPHR1/2

The knockout of candidate genes with asODNs was performed as described previously [62]. CsPHR1 and CsPHR2 were used as input sequences to obtain potential asODNs with Soligo software (Supplementary Data Table S1). For MeJA treatment, sixteen individual young shoots were soaked with ODN or asODN solution for 24 h, then MeJA was added to the ODN or asODN solution at a final concentration of 10 μM. Then young shoots that had been treated with MeJA for 24 h were collected for RT–qPCR or catechin analysis.

### Subcellular localization analysis

The cDNA sequences of CsPHR1 and CsPHR2 were cloned into pCAMBIA1300 vector with eGFP in its C-terminus by homologous recombination. 35S:CsPHR1-GFP and 35S:CsPHR2-GFP were expressed in *N. benthamiana* leaves by *Agrobacterium*-mediated transient transformation. Forty to forty-four hours after infiltration, GFP fluorescence was visualized using a confocal laser scanning microscope system (Leica Microsystems, USA).

### Transcriptional activation

The transcriptional activation assay was carried out exactly as described [64]. The ORFs of *CsPHR1*, *CsPHR2*, and *AtPHR1* were inserted into the pGBKT7 (BK) vector. BK-Lam was used as a negative control and BK-AtPHR1 as a positive control. All these vectors were transformed into AH109 with the empty vector pGADT7, grown on SD/−Leu/−Trp medium, and then selected on the SD/−Ade/−Leu/−Trp/−His medium. The primers are listed in Supplementary Data Table S1.

### Plant transformation

Columbia-0 (Col-0) was used as the wild-type. The *phr1* mutant (SALK_067629) was ordered from ABRC (Arabidopsis Biological Resource Center). For the generation of transgenic plants, *CsPHR1* and *CsPHR2* were cloned into pCAMBIA1300 vector with HA-tag in its C-terminus to generate CsPHR1-HA and CsPHR2-HA with hygromycin resistance. Then, the constructs were transformed into wild-type plants by floral dipping [65]. Transgenic lines were selected on half-strength Murashige and Skoog medium containing 50 mg/l hygromycin B.

### Yeast one-hybrid assays

The 2-kb promoter fragment of *CsANR1* and *CsMYB5c* were cloned into the pAbAi vector to generate the bait constructs pAbAi-*pCsANR1* and pAbAi-*pCsMYB5c*, respectively. After digesting with BstBI, the bait constructs were then transformed into Y1H Gold. The prey constructs were generated by introducing CsPHR1 and CsPHR2 cDNA sequences into the pGADT7 vector, and then transferred into bait-reporter yeast cells that contained *CsANR1* or *CsMYB5c* promoter fragments. Transformants were selected on SD/-Leu plates and SD/-Leu plates with aureobasidin A (AbA).

### Electrophoretic mobility shift assay

For the EMSA assay, the CsPHR1 and CsPHR2 sequences were fused with an N-terminal MBP tag (pET-HMT). Full-length CsJAZ3 was cloned into pCold TF to generate the pCold-CsJAZ3 construct with His-tag in its C-terminus. Then the recombinant MBP-tagged CsPHR1 and CsPHR2 and pCold-CsJAZ3 were transformed into *Escherichia coli* Rosetta (DE3). When the OD600 of the cultured bacterial cells reached 0.6–0.8, 1 mM isopropyl-β-d-thiogalactopyranoside (IPTG) was added to induce protein expression. As shown in Supplementary Data Fig. S6D, recombinant MBP-tagged CsPHR1 and CsPHR2 proteins and pCold-CsJAZ3 were purified using a BeaverBeads IDA-Nickel Kit (Beaver, 70 501). The Lightshift Chemiluminescent EMSA Kit (Pierce, 20 148) was used to determine the ability of CsPHRs to bind to biotin-labeled oligonucleotide probes. MBP-CsPHR1 or MBP-CsPHR2 was assayed for binding to the P1BS probes with increasing concentrations of pCold-CsJAZ3 in a total volume of 20 μl. Briefly, the binding mixture was loaded onto a 6% native polyacrylamide gel after the CsPHR proteins and biotin-labeled probes had been incubated in the binding solution for 20 min at room temperature, then the protein–DNA complex was transferred onto N+ nylon membrane (Millipore). The ChemDoc XRS System (Bio-Rad) and the enhanced chemiluminescence substrate were used to detect migration of biotin-labeled probes. Primers and biotin-labeled probes used for these constructs are listed in Supplementary Data Table S1.

### Dual luciferase reporter assay.

The CsPHR1 and CsPHR2 sequences were cloned into the pGreenII 62-SK vector, generating effector constructs. The 2-kb promoter region of *CsANR1* and 1-kb promoter region of *CsMYB5c* were cloned into a pGreenII 0800-LUC vector. pGreenII 62-SK vector was used as an internal control. These constructs were then transformed into GV3101(pSoup). The full-length cDNA of CsJAZ3 was cloned into the pCAMBIA1300 vector with Flag tag in its C-terminus. The CsJAZ3-Flag construct was then transformed into GV3101.Transient expression was conducted in leaves of *N. benthamiana* with a 1:1 mix of these *Agrobacterium* strains. A Dual-Luciferase Reporter Assay System (Promega) was used to measure the activities of the Cs*ANR1* and *CsMYB5c* promoters with the effector transcription factors or internal control. An imaging system for living plants (Lumazone PyLoN 2048B, USA) was used to capture LUC luminescence after leaves of *N. benthamiana* had been sprayed with 1 mM luciferin for 5 min in the dark.

### Bimolecular fluorescence complementation

The cDNA sequences of *CsPHR1* and *CsPHR2* were cloned into the cYFP vector and *CsSPX1* and *CsJAZ3* were cloned into the nYFP vector to obtain the CsPHR1-cYFP, CsPHR2-cYFP, CsSPX1-nYFP, and CsJAZ3-nYFP constructs, respectively. The visualization of the YFP fluorescence signal was carried out as described previously [49].

### Semi-pull-down assay

For semi-pull-down assays, transient transfected *N. benthamiana* leaves with expression of CsSPX1-Flag, CsJAZ3-Flag, or GFP-Flag were harvested and lysed using RIPA Lysis Buffer (Strong, Merck). Purified MBP-CsPHR1 and MBP-CsPHR2 on His beads (70 501; Beaver) were separately mixed with lysates of leaves harboring CsJAZ3-Flag or GFP-Flag for 1 h at 4°C. Protein complexes retained on the beads were detected with anti-Flag (GenScript) or anti-His antibody (Proteintech).

### Jasmonate measurement

Tea plants were grown under +P/–P nutrient solution for about 2 months. Young shoots were selected to analyze JA profiling. Leaf samples weighing 0.1 g were ground into a powder and then dissolved in 0.7 ml of methanol. The mixture was centrifuged at 12 000 rpm for 5 min at 4°C after being vortexed for 24 h. The supernatant was used for further analysis after centrifuging once again. As described in a previous report [62], JA profiling was carried out using liquid chromatography–tandem mass spectrometry (SCIEX 5500+).

## Accession numbers

The sequences of tea genes can be found in the Tea Plant Information Archive (TPIA) ((http://tpia.teaplants.cn/)) with the following accession numbers: *CsPHR1* (TEA002345), *CsPHR2* (TEA015819), *CsSPX1* (TEA018634), *CsPHT1;2a* (TEA029609), *CsPHT1;3a* (TEA010724), Cs*ANR1* (TEA009266), *CsANR2* (TEA022960), *CsDFR* (TEA032730), Cs*ANS* (TEA015769), *CsJAZ12*(TEA030190), and *GAPDH* (KA295375), *CsMYB5a* (KY827396), *CsMYB5b* (KY827397), *CsMYB5c* (KY827398), *CsMYB5e* (KY827400), *BnPHR1* (JN806156), and *PtPHR1* (ON075805) from the National Center for Biotechnology Information (https://www.ncbi.nlm.nih.gov/nucleotide/). The sequences of *Arabidopsis* genes were downloaded from The Arabidopsis Information Resource [TAIR - Home Page (arabidopsis.org)] with the following accession numbers: AtPHR1 (AT4G28610.1), AtPHL1 (AT5G29000), AtPHL2 (AT3G24120.2), AtPHL3 (AT4G13640.2), and AtPHL4 (AT2G20400.2). The sequences of rice genes were downloaded from (RAP-DB, http://rapdb.dna.affrc.go.jp/) with the accession numbers OsPHR1 (Os03g21240), OsPHR2 (Os07g25710), OsPHR3 (Os02g04640), and OsPHR4 (Os06g49040). The sequence data for tomato SlPHL1 (Solyc09g072830) were found from the SGN (http://solgenomics.net/). The following accession numbers can be used to access Ta-PHR1 gene sequence information in the EMBL/GenBank data libraries: *Ta-PHR1-A1* (KC218925), *Ta-PHR1-B1* (KC286910), and *Ta-PHR1-D1* (KC286911). The sequence of *CsPHL3* (Cs2g01480) was accessed from the *C. sinensis* genome database (http://citrus.hzau.edu.cn/index.php).

## Supplementary Material

Web_Material_uhae178

## Data Availability

All the data supporting the findings of this study are available in the paper and supplementary data.
