# Supplementary material for: CsPHRs-CsJAZ3 incorporates phosphate signaling and jasmonate pathway to regulate catechin biosynthesis in Camellia sinensis
Source: Hortic Res. 2024 Jun 27;11(8):uhae178. doi: 10.1093/hr/uhae178 (PMC11331543; doi:10.1093/hr/uhae178)
Supplement: Web_Material_uhae178 [file web_material_uhae178.zip › Supplementary_Material-3.docx]

**TEMPLATE FOR HR SUPPORTING INFORMATION**


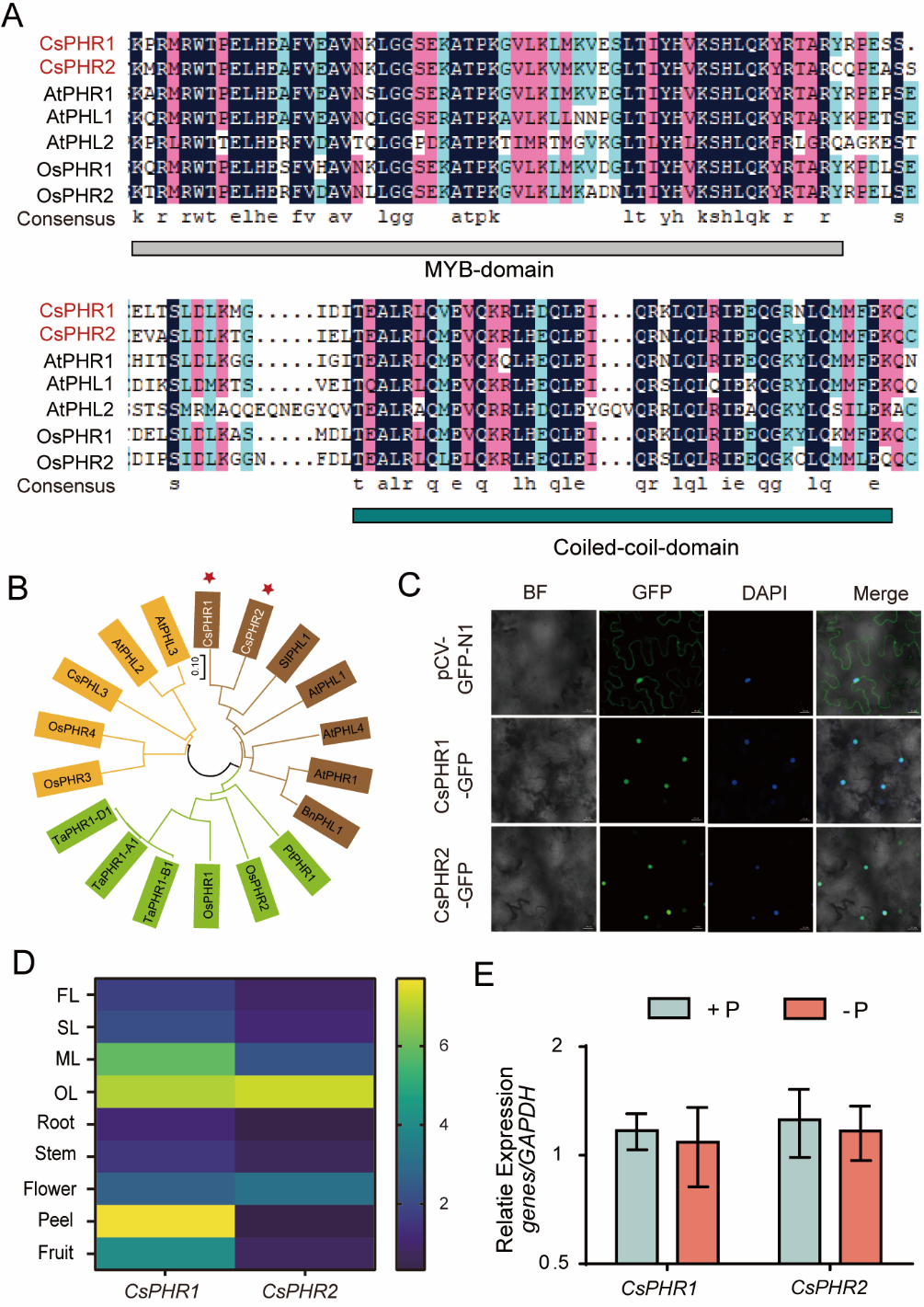


**Figure S1. Characteristic of CsPHRs. (A)** Structures of the *CsPHRs.* MYB-domain or coiled-coil-domain was marked with gray or green line, respectively. Alignment was performed using the DNAman multiple alignment tool. **(B)** Phylogenetic tree of PHR transcription factors (TFs). The phylogenetic tree between tea plant and other species were estimated by MEGA 11 using the neighbor-joining method with 1000 bootstrap replicates. The numbers indicate the confidence percentage. **(C)** Sub-cellular localization of CsPHR1 and CsPHR2. BF, blight-field image; GFP, GFP fluorescence; DAPI, 49,6-diamidino-2-phenylindole; Merge, merge of GFP and DAPI images; Different panels represent the following channels (from left to right): Bright field, GFP and all channels merged. Scale bar =20μm. **(D)** Expression of CsPHRs in different tea tissues. FL: First leaves. SL: Second leaves. ML: Mature leaves. OL: Old leaves. (**E**) Expression of *CsPHRs* in young shoots of tea that harvested upon Pi-sufficient (+P) or Pi starvation (-P) treatment for 21 days. Values are means ± SD of three biological replicates.


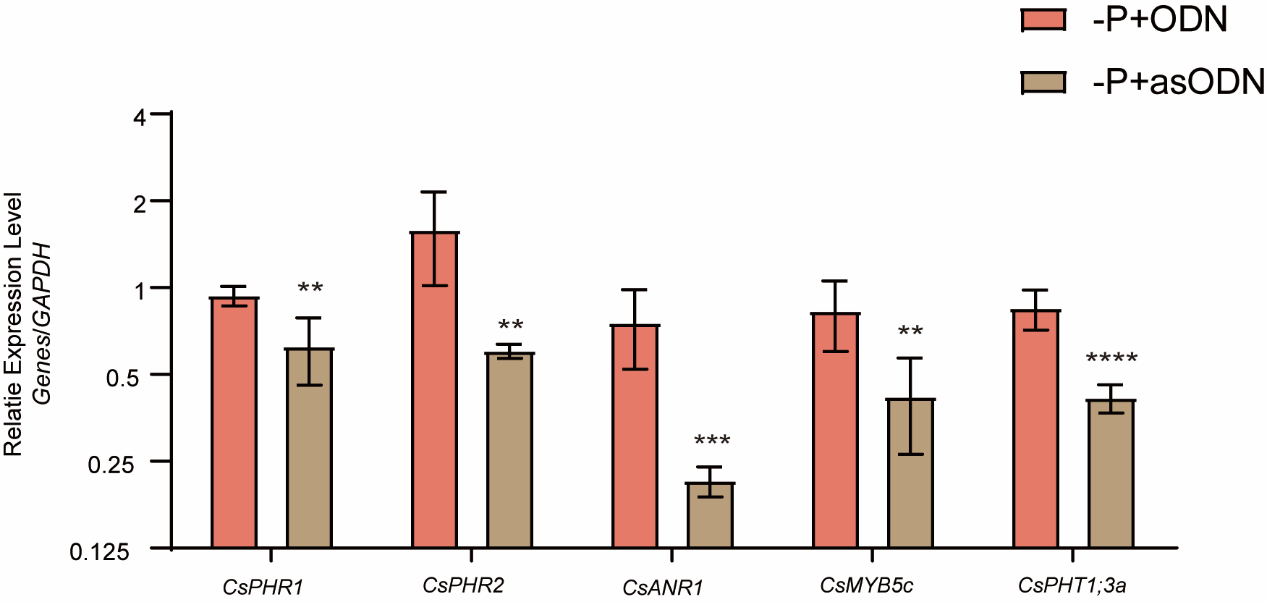


**Figure S2.** RT-qPCR analyzed genes expression of tea plants treated with ODN or asODN under Pi starvation condition. GAPDH was used as an internal control. Values represent means ± SEM (n=6). ****P < 0.0001; ***P < 0.001; **P < 0.01. Student’s t-test compared with ODN-treated samples.


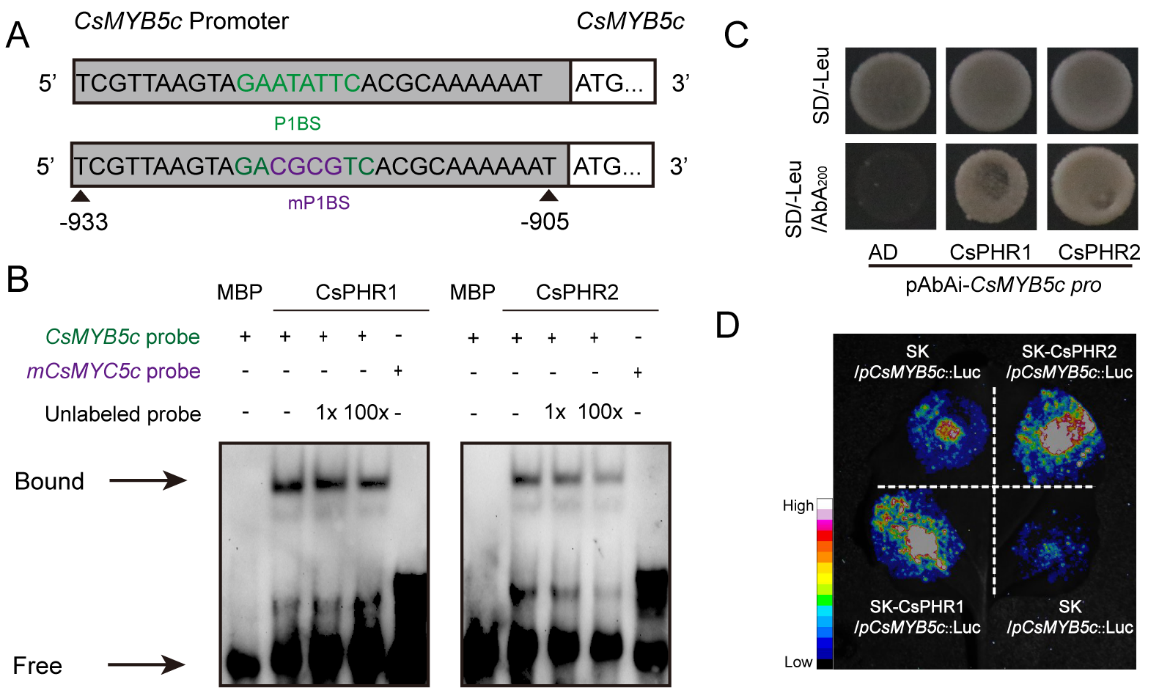


**Figure S3. CsPHRs regulated expression of *CsMYB5c* by binding the P1BS element directly.** **(A)** Diagram of the wild-type P1BS element and four base-mutated P1BS (mP1BS) in the *CsMYB5c* promoter. **(B)** Y1H analysis of CsPHRs binding the promoter of *CsMYB5c*. Empty pGADT7 vector was used as negative control. Yeast cells containing different plasmid combinations were grown on the selective medium SD-Leu with 100μm Aureobasidin A (AbA). **(C)** EMSA results indicated CsPHR proteins bind to the *CsMYB5c* promoter. Arrows show CsPHRs-bound or free DNA. The competitive protein-DNA binding assay was performed with an increasing amount of unlabeled DNA probe (1-fold, 10-fold and 20-fold) **(D)** Luciferase activity assay in *N*. *benthamiana* leaves. Luciferase intensity was imaged at 48h after infiltration.


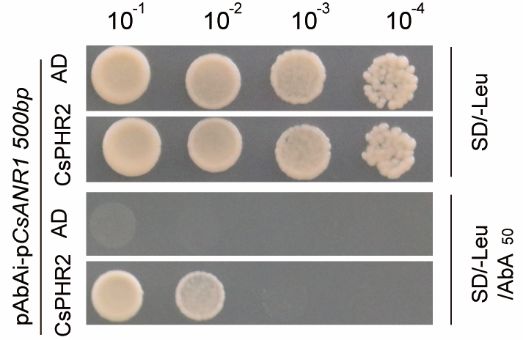


**Figure S4.**Y1H analysis of CsPHR2 binding the 0.5-kb length promoter of *CsANR1* that containing P1BS. Empty pGADT7 vector was used as negative control. Yeast cells containing different plasmid combinations were grown on the selective medium SD-Leu with 50μm Aureobasidin A (AbA).


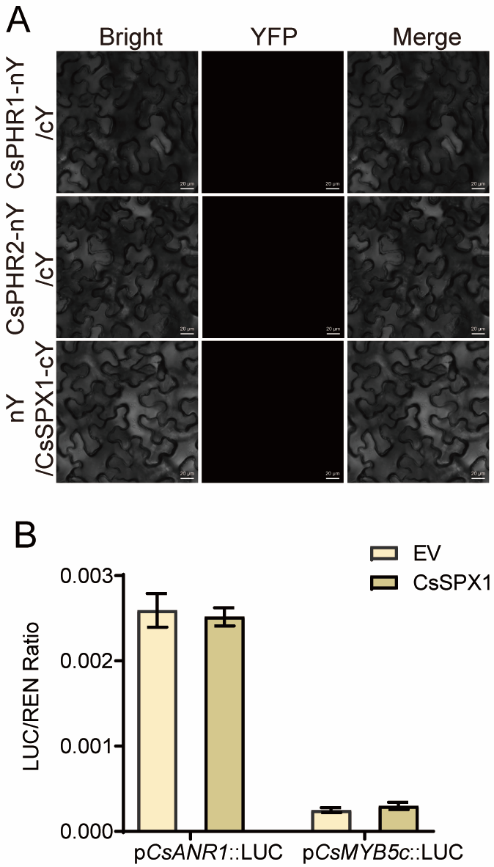


**Figure S5. (A)** Negative control of interaction analysis between CsSPX1 and CsPHR1/2 by BiFC. Confocal images of N. *benthamiana* epidermal cells expressing different construct combinations as indicated were shown. (Scale bar: 20 μm). **(B)** Luciferase activity assay in *N*. *benthamiana* leaves.


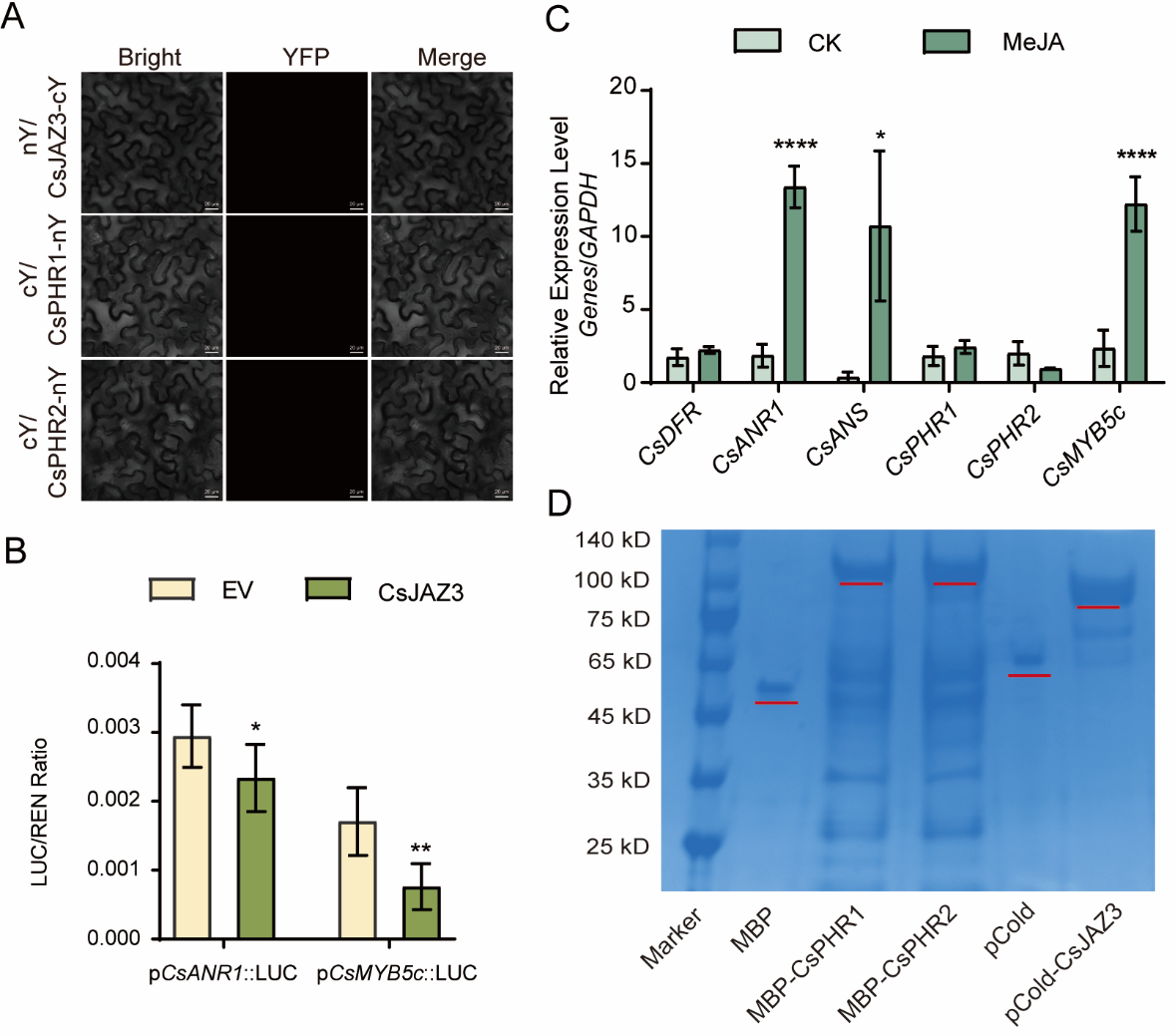


**Figure S6.** **(A)** Negative control of interaction analysis between CsPHR1/2 and CsJAZ3 by BIFC. Confocal images of N. *benthamiana* epidermal cells expressing different construct combinations as indicated were shown. (Scale bar: 20 μm). **(B)** Relative transcript levels of genes involved in catechins biosynthesis under MeJA treatment by RT-qPCR. GAPDH was used as an internal control. Values represent means ± SEM (n=3). ****P < 0.0001; ***P < 0.001; **P < 0.01. **(C)** Luciferase activity assay in *N*. *benthamiana* leaves. **(D)** SDS–PAGE of purified recombinant proteins. The correspondent proteins were marked with red lines.


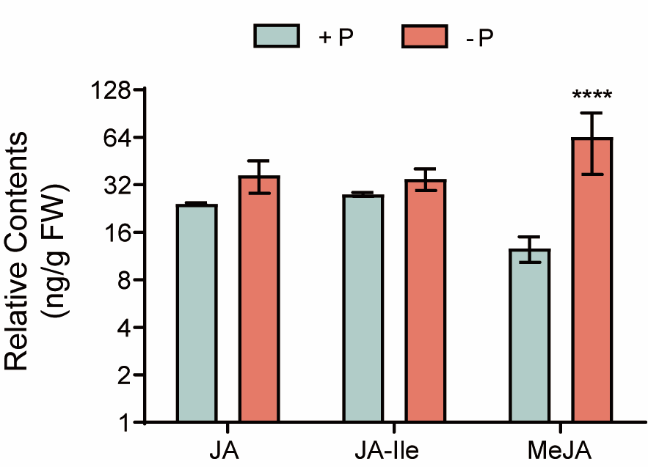


**Figure S7.** Pi starvation induces MeJA production. Error bars = SD± SE of three biological replicates. ****P < 0.0001, Student's t-test.

**Table S1. Constructs and Primers Used in This Study.**

| Destination | Primer sequence(5’-3’) |
| --- | --- |
| For RT–qPCR | |
| qGADPH | F: TTGGCATCGTTGAGGGTCT |
|  | R: CAGTGGGAACACGGAAAGC |
| qCsPHR1 | F: TGGAGAGCCTTGTGGTGTTC |
|  | R: TCTGAAGGTGGCTCTTCACAT |
| qCsPHR2- | F: GCTGAAGGCCTCATCATCGT |
|  | R: CCACAACGTCATTTGCCTCG |
| qCsPHT1;2b | F: CACGGGTTTCACTTGCTTGG |
|  | R: CCGTAAACCAGTAGCCAGGG |
| qCsPHT1;3 | F: GCAAACAAGAAGACTCGCGG |
|  | R: GGGAAGTGCTCCAACCATCA |
| qCsANS | F: CATAGACTCGGAGGTCGAGGA |
|  | R: CGCTATCCCATGATTCACCA |
| qCsANR1 | F: GCAGCGTGGAAATTTGCTGA |
|  | R: ATGTGCGCACCACAAACATC |
| qCsANR2 | F: TTGGTCTTGCCATGTCCTTG |
|  | R: TGGAGATTGAGCCTGACAGC |
| qCsDFR | F: CACATCCTCTGCTGGAACTG |
|  | R: ATGCTGCTTTCTCTGCCAAT |
| qCsMYB5a | F: CGGGCCGAACAGACAATGAA |
|  | R: GACAGTGGCTGGCCTTTTTG |
| qCsMYB5b | F: AGTTTTCATCCGACCGGACC |
|  | R: ACAGTGTCAGTAGTGTCGCC |
| qCsMYB5c | F: GGAAGAGTTGCAGGCTAAGGT |
|  | R: TGTAGGTTGGGGTCGGAAT |
| qCsMYB5e | F: ACAATGACCACTACCGCTCC |
|  | R: CGGTGGTTTGGTCAGGTTTG |
| qAtF3’H | F: ACACCGATGGAGACTGTTGAGAA |
|  | R: GCGTTAGCGTTCCAACCTCTTC |
| For Silencing of CsPHR1/2 | |
| ODN | CCTTTATTTCTCAGTCATCAA |
| asODN | TTGATGACTGAGAAATAAAGG |
| For subcellular localization and plant transformation | |
| pCV-CsPHR1-HA | F: GACTCTAGACCCCTGGGATCCATGGAAGCTCGCCCTGCTT |
|  | R: CGCCCTTGCTCACCATGTCGACTTCACCTGGTTTTGCACGCC |
| pCV-CsPHR2-HA | F: GACTCTAGACCCCTGGGATCCATGGAAGCACGCCCTGCAAG |
|  | R: CGCCCTTGCTCACCATGTCGACTTCATCTGCTCTCGCGCGCT |
| For transcriptional activation | |
| BK-CsPHR1 | F: AGGATTTCAGAATTCGGATCCATGGAAGCTCGCCCTGCTTT |
|  | R: TGCCTGCAGGTCGACTCTAGATTATTCACCTGGTTTTGCAC |
| BK-CsPHR2 | F: AGGATTTCAGAATTCGGATCCATGGAAGCACGCCCTGCAAG |
|  | R: TGCCTGCAGGTCGACTCTAGATTATTCATCTGCTCTCGCGC |
| BK-AtPHR1 | F: AGGATTTCAGAATTCGGATCCATGGAGGCTCGTCCAGTTCA |
|  | R: TGCCTGCAGGTCGACTCTAGATCAATTATCGATTTTGGGAC |
| For Y1H | |
| pAbAi-CsANR1pro | F:TTGAATTCGAGCTCGGTACCTGGTTGTACTAGATTAGACA |
|  | R:ACCGCGGTGGCGGCCGCGGCTTTTCTCACTTTGAGAG |
| pAbAi-CsMYB5cpro | F:AAAATGATGAATTGAAAAGCTTTAAAGTTTTCTTTTTTCGTTGATC |
|  | R:GAGCACATGCCTCGAGGTCGACTTTGTTTGTTTCTCAGGAAAGTCAG |
| AD-CsPHR1 | F:GACGTACCAGATTACGCTCATATGATGGAAGCTCGCCCTGCTTT |
|  | R:GCAGCTCGAGCTCGATGGATCCTTCACCTGGTTTTGCACGCC |
| AD-CsPHR2 | F:GACGTACCAGATTACGCTCATATGATGGAAGCACGCCCTGCAAG |
|  | R:GCAGCTCGAGCTCGATGGATCCTTCATCTGCTCTCGCGCGCT |
| For EMSA | |
| MBP-CsPHR1 | F: CTGTATTTTCAGGGCCATATGATGGAAGCTCGCCCTGCTTT |
|  | R: ACGGAGCTCGAATTCGGATCCttaTTCACCTGGTTTTGCAC |
| MBP-CsPHR2 | F:CTGTATTTTCAGGGCCATATGATGGAAGCACGCCCTGCAAG |
|  | R:ACGGAGCTCGAATTCGGATCCttaTTCATCTGCTCTCGCGC |
| pCold-CsJAZ3 | F: GTATCGAAGGTAGGCATATGATGTCGAGTTCGTCTGGTTC |
|  | R: ACAAGCTTGAATTCGGATCCCTACAAATGGTGCTCAAACT |
| CsANR1 prob | F:TGGTTGTACTAGATTAGACATTGCATATGCAGTGGGAGTGGTTAGTAAAGTAATC |
|  | R:GATTACTTTACTAACCACTCCCACTGCATATGCAATGTCTAATCTAGTACAACCA |
| mCsANR1 prob | F:TGGTTGTACTAGATTAGACATTGCCGCGGCAGTGGGAGTGGTTAGTAAAGTAATC |
|  | R:GATTACTTTACTAACCACTCCCACTGCCGCGGCAATGTCTAATCTAGTACAACCA |
| CsMYB5c prob | F:CCGTTTATATTTTAGGACTCGTTAAGTAGAATATTCACGCAAAAAATAAGATTATC |
|  | R:CCGTTTATATTTTAGGACTCGTTAAGTAGAGCGCTCACGCAAAAAATAAGATTATC |
| mCsMYB5c prob | F:CCGTTTATATTTTAGGACTCGTTAAGTAGAGCGCTCACGCAAAAAATAAGATTATC |
|  | R:CCGTTTATATTTTAGGACTCGTTAAGTAGAGCGCTCACGCAAAAAATAAGATTATC |
| For dual luciferase reporter assay | |
| pGreen-pCsANR1 | F: GCAGCCCGGGGGATCCTGGTTGTACTAGATTAGACA |
|  | R: ACCGCGGTGGCGGCCGCGGCTTTTCTCACTTTGAGAG |
| pGreen-pCsMYB5c | F: cttgatatcgaattcctgcagTAAAGTTTTCTTTTTTCGTTGATC |
|  | R: atgtttttggcgtcttccatGGTATCACCATGTAGGGCACAC |
| SK-CsPHR1 | F: cgctctagaactagtggatccATGGAAGCTCGCCCTGCTTT |
|  | R: cttgatatcgaattcctgcagTTCACCTGGTTTTGCACGCC |
| SK-CsPHR2 | F: cgctctagaactagtggatccATGGAAGCACGCCCTGCAAG |
|  | R: cttgatatcgaattcctgcagTTCATCTGCTCTCGCGCGC |
| CsSPX1-Flag | F: CgACgACAAgACCgTCACCATGGCCATGGAGGCCAGTGA |
|  | R: gAggAgAagAgCCgTCgTAGGGATTTCTTCCATGCCG |
| CsJAZ3-Flag | F: CgACgACAAgACCgTCACCatgATGTCGAGTTCGTCTGGTTC |
|  | R: ggAgAagAgCCgTCgCAAATGGTGCTCAAACTGCA |
| For BIFC | |
| CsPHR1-cYFP | F: CgACgACAAgACCgTCACCatgATGGAAGCTCGCCCTGCTTT |
|  | R: ggAgAagAgCCgTCgTTCACCTGGTTTTGCACGCC |
| CsPHR2-cYFP | F: CgACgACAAgACCgTCACCatgATGGAAGCACGCCCTGCAAG |
|  | R: ggAgAagAgCCgTCgTTCATCTGCTCTCGCGCGCT |
| CsSPX1-nYFP | F: CgACgACAAgACCgTCACCATGGCCATGGAGGCCAGTGA |
|  | R: gAggAgAagAgCCgTCgTAGGGATTTCTTCCATGCCG |
| CsJAZ3-nYFP | F: CgACgACAAgACCgTCACCatgATGTCGAGTTCGTCTGGTTC |
|  | R: ggAgAagAgCCgTCgCAAATGGTGCTCAAACTGCA |
